# B-line quantification: comparing learners novice to lung ultrasound assisted by machine artificial intelligence technology to expert review

**DOI:** 10.1186/s13089-021-00234-6

**Published:** 2021-06-30

**Authors:** Frances M. Russell, Robert R. Ehrman, Allen Barton, Elisa Sarmiento, Jakob E. Ottenhoff, Benjamin K. Nti

**Affiliations:** 1 Department of Emergency Medicine, Indiana University School of Medicine, 720 Eskenazi Ave, FOB 3rd Floor, Indianapolis, IN 46202 USA; 2 Department of Emergency Medicine, Wayne State University School of Medicine, 4021 St Antoine Ave, Suite 6G, Detroit, MI 48201 USA; 3 Boone County Emergency Physicians, Zionsville, IN 46077 USA

**Keywords:** Artificial intelligence, Point-of-care ultrasound, Lung ultrasound, Acute heart failure, Novice learner

## Abstract

**Background:**

The goal of this study was to assess the ability of machine artificial intelligence (AI) to quantitatively assess lung ultrasound (LUS) B-line presence using images obtained by learners novice to LUS in patients with acute heart failure (AHF), compared to expert interpretation.

**Methods:**

This was a prospective, multicenter observational study conducted at two urban academic institutions. Learners novice to LUS completed a 30-min training session on lung image acquisition which included lecture and hands-on patient scanning. Learners independently acquired images on patients with suspected AHF. Automatic B-line quantification was obtained offline after completion of the study. Machine AI counted the maximum number of B-lines visualized during a clip. The criterion standard for B-line counts was semi-quantitative analysis by a blinded point-of-care LUS expert reviewer. Image quality was blindly determined by an expert reviewer. A second expert reviewer blindly determined B-line counts and image quality. Intraclass correlation was used to determine agreement between machine AI and expert, and expert to expert.

**Results:**

Fifty-one novice learners completed 87 scans on 29 patients. We analyzed data from 611 lung zones. The overall intraclass correlation for agreement between novice learner images post-processed with AI technology and expert review was 0.56 (confidence interval [CI] 0.51–0.62), and 0.82 (CI 0.73–0.91) between experts. Median image quality was 4 (on a 5-point scale), and correlation between experts for quality assessment was 0.65 (CI 0.48–0.82).

**Conclusion:**

After a short training session, novice learners were able to obtain high-quality images. When the AI deep learning algorithm was applied to those images, it quantified B-lines with moderate-to-fair correlation as compared to semi-quantitative analysis by expert review. This data shows promise, but further development is needed before widespread clinical use.

## Background

Lung ultrasound (LUS) for the evaluation of pulmonary edema in acute heart failure (AHF) has become standard care in many emergency departments (ED) and intensive care settings [1]. Ultrasound can be used by practitioners across a broad range of expertise to evaluate the lungs for the presence of B-lines, indicating pulmonary edema or loss of lung aeration [2]. Presence of B-lines is highly specific for the presence of interstitial and alveolar fluid. In the evaluation of a patient with dyspnea, this can rapidly alter the differential as well as dictate initial management [3]. Delays in recognizing pulmonary congestion can delay initiation of appropriate treatments and increase the likelihood of adverse events.

The workup of AHF has traditionally centered on identifying radiographic and physical exam findings consistent with fluid overload such as pretibial edema, rales, hepatojugular reflux, and pulmonary edema on chest X-ray. The most recent 2019 guidelines from the American College of Cardiology continue to emphasize estimation of fluid status and therapy centered on decongestion in AHF [4]. However, even in AHF without total body fluid overload (e.g., Sympathetic Crashing Acute Pulmonary Edema), identifying pulmonary edema is critical in expediting diagnosis and management [5]. LUS has consistently been shown to identify extravascular lung water with greater accuracy than chest X-ray and has the advantage of being performed rapidly and repeatedly at the bedside [6, 7]. However, LUS relies heavily on clinician competence in obtaining and interpreting images. The use of artificial intelligence (AI) software packages embedded in ultrasound systems to identify and quantify B-lines has the potential to offer these same benefits to novice learners without extensive ultrasound training.

While automated LUS image analysis has been described [8, 9], no prior study has investigated how existing AI B-Line quantification packages perform when used in this putatively beneficial scenario. Therefore, the goal of this study was to compare B-line quantification from an AI software package to those of an expert reviewer, using images acquired by novice learners on ED patients with AHF. Secondarily, we assessed image quality.

## Methods

### Study design and patient selection

This was a prospective observational study performed from December 2018 to March 2020 at two urban academic EDs, each of which supports a 3-year Emergency Medicine (EM) residency and serves as a site for medical student EM rotations. The study was approved by the institutional review board at each site (protocol #1809442708).

Patients > 18 years-of-age who were able to provide written informed consent were eligible for inclusion when the following criteria were met: subjective report of dyspnea at rest or with minimal exertion, a clinical diagnosis of AHF by the treating provider, and clinical signs of volume overload (pulmonary edema on chest X-ray, jugular venous distension, pulmonary rales/crackles on chest auscultation, or bilateral lower extremity edema). Patients with a temperature > 38.6° Celsius, history of interstitial lung disease, or suspected acute lung injury/acute respiratory distress syndrome were excluded. Demographics, past medical history, New York Heart Association (NYHA) symptom classification, and B-type natriuretic peptide level were collected for each patient. Study data were collected and managed using REDCap electronic data capture tools.

### Novice learner training and lung ultrasound protocol

Novice learners consisted of medical students and EM residents. In order to be able to enroll patients in the study, novice learners first participated in a 30-min training session lead by a study investigator at each site that consisted of a brief lecture covering the etiology and appearance of B-lines on LUS as well as image acquisition technique. After the initial lecture, novice learners completed proctored hands-on scanning of patients with pulmonary edema. Each participating learner completed a data collection form recording their level of training, total number of US examinations of any modality performed, and total number of LUS examinations performed previously.

LUS examinations were performed in accordance with a previously published protocols [10]. Briefly, patients were scanned supine with head-of-bed elevation as close to 45 degrees as their comfort would allow. All exams were performed using a GE Venue ultrasound system (GE Healthcare, Milwaukee, WI) with a curvilinear probe in the “Lung” setting. Depth was set to 18 cm, and the probe was placed in a horizonal orientation (indicator to patient’s right side) in 4 zones per hemi-thorax. Tissue harmonics and compounding were turned off, speckle reduction minimal, and the focal zone was defaulted to just deep to one centimeter to be close to the pleural line. For each lung zone, a 6-s video clip was stored. Novice learners were aware of the study purposes and were not blinded to patient physical appearance. The same patient could be scanned by more than one learner (if the patient was willing), but only one learner was present at a time when serial examinations were performed.

### B-line quantification

**Fig. 1:**
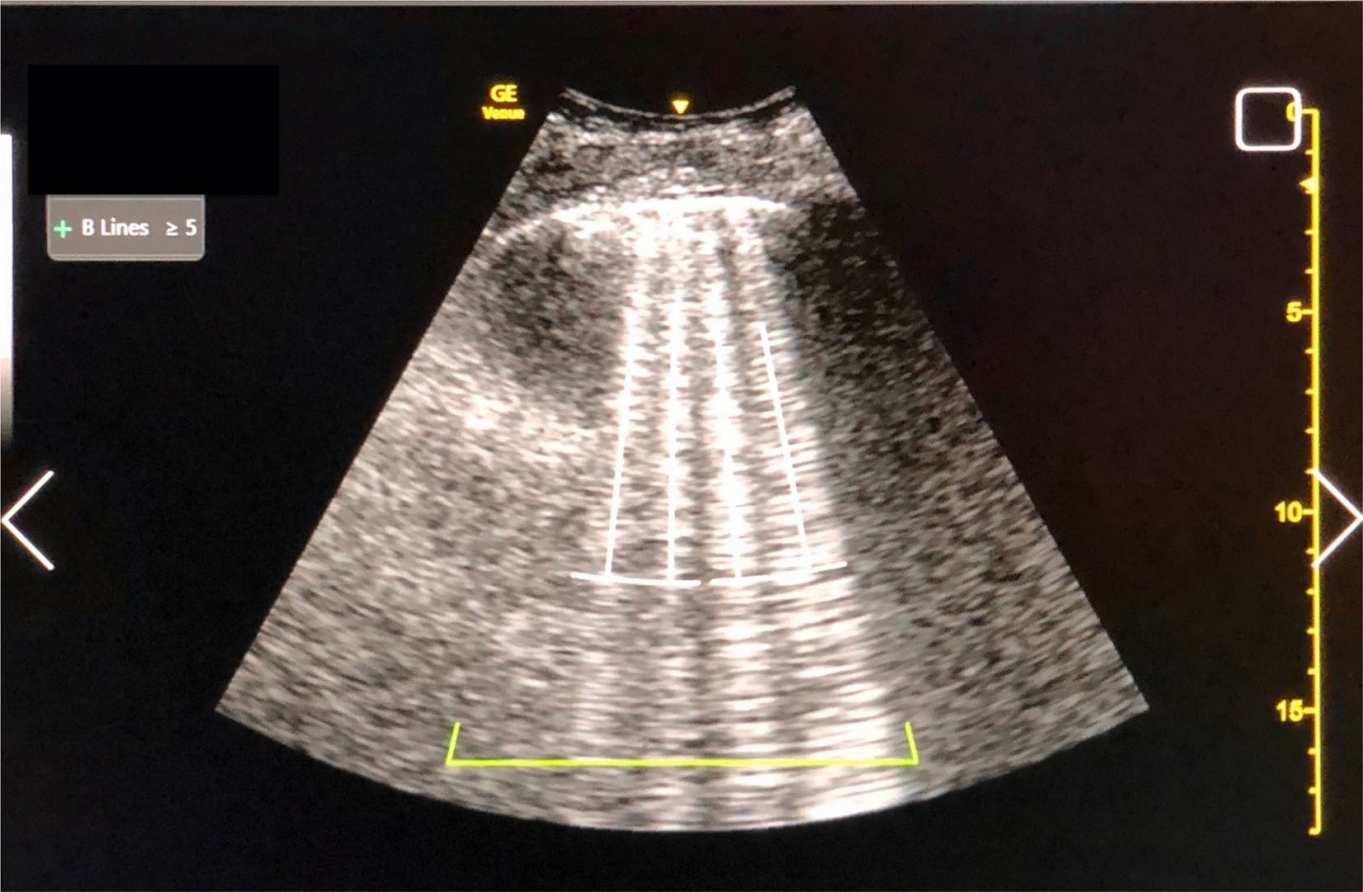
Lung ultrasound image of B-lines using machine AI for quantification. This image shows the zone analyzed (green line at bottom) and demonstrates ≥ 5 B-lines

B-line counts by novice learners were obtained by using the “Auto B-Line” AI feature on the US system. This feature was turned off during image acquisition and the counts were obtained offline once the examination was completed. This was to allow later blinded quantification by an expert. The AI settings were configured to a “scan across rib spaces” setting, which automatically counts B-lines in the central two-thirds of the image rather than across the entire screen. The software package counts the maximum number of B-lines visualized during the clip in integers from 0 to 5, or ≥ 5 if there are more than 5 discrete B-lines or confluence of B-lines occupying > 50% of the pleural line (Fig. 1). Clips where no pleural line was visible on the LUS image were excluded from analysis. Clips where bowel or rib was imaged, and no pleural line was seen, were excluded from AI analysis but were included in the image quality analysis.

The criterion standard for B-line counts was manual counts performed by a point-of-care ultrasound expert physician reviewer with extensive experience in LUS research. They were blinded to AI counts and patient information. De-identified clips were exported from the US system and interpreted offline, using the same counting method. The expert reviewer determined image quality using a 5-point scale (1: pleural line not in view, 2: small part of pleural line in view with technical flaws, 3: moderate amount of pleural line in view with technical flaws, 4: pleural line in full view with small technical flaws, 5: pleural line in view with excellent image quality). A score > 3 was pre-determined to be good image quality. A randomized subset of LUS videos were assessed for B-line quantification and image quality by a second expert reviewer, with extensive LUS experience, to assess for expert agreement. This second expert was not involved in the study design or training. They were blinded to clinical data, site, and expert and AI quantification.

### Statistical analysis

We reported frequencies and percentages for categorical variables and median (minimum–maximum) for continuous variables. Data were compared using the Wilcoxon test and Spearman’s correlation for determining interclass correlation. We performed all statistical analysis using SAS, Version 9.4 (SAS Institute, Cary, NC). On individual scans, machine AI was considered to be in agreement with the expert if counts were within 1 B-line. Intraclass correlation (ICC) coefficients were used to determine agreement between experts for B-line quantification and image quality.

## Results

Eighty-seven LUS examinations (696 lung zones) were performed on 29 individual patients. Out of 696 lung zones assessed, 611 (88%) images were used in the B-line quantification analysis. To be eligible for the B-line quantification analysis, the images had to show a pleural line interface. Thus, 85 images were excluded: 4 images were either not saved properly or not acquired, and 81 images did not include a pleural interface. For example, if the image only included a heart in view it was excluded from analysis, which is common in left zone 2. Additionally, if the patient had a large pleural effusion and or pneumonia these scans were excluded from analysis. If the learner only captured bowel or ribs these images were also excluded from quantification analysis (*n* = 28).

Out of 696 lung zones assessed, 639 (92%) were analyzed for image quality. The 28 videos, where images were inadequately acquired (i.e., only bowel or rib was visualized), were excluded from quantification analysis, but included in the image quality analysis.

The median age of included patients was 65, 55% of patients were male, and the median body mass index was 28.2. Twenty-two (76%) patients had a history of heart failure, 14 with a history of systolic heart failure, and 8 with diastolic heart failure. A majority of patients were NYHA class 2. Median initial B-type natriuretic peptide level was 576. Sixteen (55%) patients were admitted to a medical floor with cardiac monitoring, 4 (13%) were admitted to the intensive care unit and 5 (17%) were discharged from the emergency department. See Table 1 for patient demographics.

**Table 1:** Patient demographics, *n* = 29, n (%)

Age, median (Min.–Max.)	65.0 (31–90)
Gender, male	16 (55.2)
Race	
Black	12 (41.4)
Hispanic	1 3.5)
White	16 (55.2)
Height in cm, median (Min.–Max.)	170.2 (149.9–193.0)
BMI, median (Min.–Max.)	28.2 (18.8–58.4)
Heart failure history	
New onset	7 (24.1)
Systolic HF	14 (48.3)
Diastolic (HFpEF)	8 (27.6)
NYHA, symptom classification	
Class 1	1 (14.4)
Class 2	17 (58.6)
Class 3	7 (24.1)
Class 4	4 (13.8)
Initial BNP level, median (Min.–Max.)	576.0 (26.0–5,000.0)
Disposition from ED	
Admit (non ICU)	16 (55.17)
ICU	4 (13.79)
Observation	4 (13.79)
Discharge	5 (17.24)

*BMI* body mass index, *ICU* intensive care unit, *HF* heart failure, *HFpEF* heart failure with preserved ejection fraction

There were 51 learners that completed the LUS examinations: 18 were medical students, 21 interns, and 12 residents. The level of ultrasound experience varied with the median number of prior LUS ranging from 1 to 10 overall. See Table 2 for learner demographics.

**Table 2:** Learner demographics, *n* = 51

	MS4 *n* = 18	Intern *n* = 21	Resident *n* = 12
Level of US experience	Some (none–moderate)	Some (some–large)	Large (moderate–large)
LUS prior	1–10 (0–25)	11–25 (0–50)	25–50 (25- > 100)

Of the 611 lung zones analyzed, 281 (46%) had 0–2 B-lines, 105 (17%) had 3–4 B-lines, and 225 (37%) had 5 or greater B-lines. Intraclass correlation between ultrasound machine AI and expert read was 0.56 (0.51–0.62) overall. See Table 3 for correlation by zone, and median read by machine AI and expert by lung zone. Correlation was highest in the lateral zones (right zone 3, 4 and left zone 3), and lowest in the left anterior lung zones.

**Table 3:** ICC between machine AI and expert, *n* = 611

Zone	GE machine, median (Min.–Max.)	Expert, median (Min.–Max.)	Spearman correlation (95% CI)
Right zone 1	5.0 (0.0–5.0)	3.0 (0.0–5.0)	0.57 (0.42–0.70)
Right zone 2	4.0 (0.0–5.0)	3.0 (0.0–5.0)	0.46 (0.27–0.62)
Right zone 3	5.0 (0.0–5.0)	2.5 (0.0–5.0)	0.65 (0.51–0.76)
Right zone 4	5.0 (0.0–5.0)	4.0 (0.0–5.0)	0.77 (0.66–0.85)
Left zone 1	4.0 (0.0–5.0)	3.0 (0.0–5.0)	0.40 (0.21–0.57)
Left zone 2	4.0 (0.0–5.0)	2.0 (0.0–5.0)	0.32 (0.05–0.55)
Left zone 3	4.0 (0.0–5.0)	2.5 (0.0–5.0)	0.60 (0.44–0.72)
Left zone 4	5.0 (0.0–5.0)	5.0 (0.0–5.0)	0.54 (0.35–0.69)
Overall ICC	–	–	0.56 (0.51–0.62)

Intraclass correlation between machine AI and expert by zone and overall. Data are based off of 611 images

Overall image quality was a median of 4, range 1 to 5, with all zones having a median of 4 or 5; see Table 4. ICC between machine AI and expert based on image quality was 0.58 (confidence interval [CI] 0.52–0.64) for scans with a quality rating of 4 or 5, and 0.41 (CI 0.37–0.53) for scans with a quality rating of 1 through 3, see Table 5. ICC between faculty experts after blinded review of 33% of images was 0.82 (CI 0.73–0.91) for B-line quantification and 0.65 (CI 0.48–0.82) for image quality.

**Table 4:** Image quality by zone and overall, *n* = 639

Zone	Median (Min.–Max.)
Right zone 1	5.0 (3.0–5.0)
Right zone 2	4.0 (1.0–5.0)
Right zone 3	4.0 (1.0–5.0)
Right zone 4	4.0 (1.0–5.0)
Left zone 1	5.0 (2.0–5.0)
Left zone 2	4.0 (1.0–5.0)
Left zone 3	4.0 (1.0–5.0)
Left zone 4	4.0 (1.0–5.0)
Overall image quality	4.0 (1.0–5.0)

Image quality by zone and overall. Data are based off of 639 images

**Table 5:** ICC between machine AI and expert by image quality, *n* = 611

Zone	Overall image quality (1–3) Spearman correlation (95% CI)	Overall image quality (4–5) Spearman correlation (95% CI)
Right zone 1	0.68 (-0.16–0.95)	0.58 (0.41–0.71)
Right zone 2	0.17 (-0.31–0.58)	0.56 (0.36–0.71)
Right zone 3	0.55 (0.24–0.76)	0.62 (0.42–0.76)
Right zone 4	0.53 (0.11–0.79)	0.67 (0.49–0.80)
Left zone 1	0.06 (-0.51–0.59)	0.48 (0.27–0.64)
Left zone 2	0.50 (-0.19–0.86)	0.27 (-0.04–0.54)
Left zone 3	0.41 (0.04–0.68)	0.51 (0.29–0.69)
Left zone 4	0.22 (-0.22–0.59)	0.63 (0.42–0.78)
Overall correlation	0.41 (0.37–0.53)	0.58 (0.52–0.64)

Intraclass correlation between machine AI and expert by image for each zone and overall. Data are based off of 611 images

## Discussion

LUS B-line quantification is a useful tool for diagnosis, prognosis, and monitoring response to treatment in patients with AHF [10–13]. European Society of Cardiology expert consensus guidelines support the use of LUS in the management of AHF [14]. LUS image acquisition and interpretation is highly dependent on the skill of the operator. Machine AI with technology to automatically quantify B-lines has the potential to decrease inter-operator variability and if this technology works it could allow novice learners the ability to count B-lines and incorporate findings into their clinical decisions in patients with AHF.

The results of our study suggest that after limited training, learners with some to no prior LUS experience were able to generate high-quality images. Machine AI was able to quantify B-lines using these images with fair correlation when compared to an expert reviewer. This data suggests that further AI technology development is necessary to improve the algorithm to achieve good correlation. If successful, this has great implications clinically as a clinician or even a non-clinician could track treatment progress in patients with AHF to determine response to treatment, guide additional therapy, and determine when a patient is decongested and ready for hospital discharge [2].

Overall machine AI tended to overcount the number of B-lines when compared to the expert in every lung zone expect left zone 4, where median counts were equal. Interestingly, the lateral zones had higher correlation with expert reads than the anterior lung zones. Anterior lung zones are generally easier to acquire compared to the lateral lung zones which require the probe be positioned in an oblique manner to stay within the rib space, which can be challenging for a novice learner to orient the probe. In addition, lateral lung zone image acquisition is difficult in obese patients.

The left anterior lung zones 1 and 2 had the lowest correlation 0.40 and 0.32, respectively. These zones also had the lowest number of assessments as the heart commonly sits in view and can make it tough to view the pleural line. If any portion of the pleural line was obtained these images were included in analysis. It is possible that the machine AI overcounted B-lines in these zones secondary to cardiac motion.

We found that after a 30-min training session, learners both novice and those with limited LUS experience were able to obtain high-quality images on the vast majority of patients. However, it is important to note that 28 (4%) images were inadequately acquired, where the learner was unable to obtain pleural line. In these instances, it was common for the learner to image below the diaphragm or over a rib. Bowel gas can appear similar to lung artifacts on US and could easily be confused by a novice learner. Additionally, rib with shadow can appear similar to pleural line, especially when viewing in a horizontal orientation. While this may have been expected in novice learners, the proportion of these inadequate images was quite low supporting that a brief training is sufficient. The importance of image quality is demonstrated by the fact that correlation between AI and expert counts increased as image quality improved—from 0.41 with lower quality images to 0.58 with higher-quality images (rated as a 4 or 5).

To our knowledge, this was the largest study assessing novice learners’ ability to use machine AI to objectively quantify LUS B-lines. Prior literature has found that machine-assisted quantification of LUS artifacts generally performs well, but studies are small. Corradi et al. [15] evaluated 32 patients with suspected community-acquired pneumonia, comparing quantitative ultrasound to chest X-ray and computed tomography as the gold standard. They found quantitative ultrasound to have high sensitivity, specificity, and diagnostic accuracy, outperforming chest X-ray and visual ultrasonography for the diagnosis of community-acquired pneumonia. Although this was a small study, evaluating pneumonia and not B-line quantification, they were able to show machine AI’s ability to accurately detect lung artifacts.

Brusasco et al. [8] studied 12 intensive care unit patients with acute respiratory distress, comparing an automated quantitative scoring system for B-lines with semi-quantitative measurements of extravascular lung water using thermo-dilution. They found computer-aided B-line quantification on LUS had a strong correlation with extravascular lung water (R^2^ = 0.57). This was a pilot study limited by a small sample size and single sonographer. Additionally, the B-line analysis was performed in post-processing, limiting the real-time application of the technology.

Corradi et al. [16] assessed computer-assisted LUS B-line quantification in 48 ventilated cardiac surgery patients, compared to pulmonary capillary wedge pressure or extravascular lung water assessments using thermo-dilution. They found high correlations between quantitative LUS and pulmonary congestion. This study differs from ours in that it included ventilated cardiac patients, different standards were used for comparison, and all images were obtained by the same operator.

The data from these studies and our study suggest that use of AI software to identify clinically useful LUS artifacts—including B-line quantification—shows promise, but further development is needed before widespread use. Future studies should be aimed at further refining this technology and prospectively assessing a larger number of patients and novice learners in diverse clinical environments, with careful attention to the impact of image quality on algorithm performance.

There are several limitations to consider. This was overall a relatively small study, although there were a large number of learners. Previously published methods of LUS B-line assessment have used differing protocols and semi-quantitative methods [17]. For this study, we used an 8-zone protocol and compared an automatic quantitative method to a semi-quantitative method by one expert. Our criterion standard was expert review using a semi-quantitative method. While this is currently used in clinical practice and has a high correlation with extravascular lung water (EVLW) [18], the true quantity of EVLW present remains unknown as there was no direct measurement thereof. In addition, we found high correlation between experts.

The machine AI software only has the ability to count 0–4 and ≥ 5 B-lines within one lung zone. Semi-quantitative methods typically use a scale of 0 through 10, or 0 through 20. From a clinical standpoint, a count of ≥ 5 B-lines within a lung zone by the US machine would be significant for a large amount of extravascular lung water, and thus clinically significant pulmonary edema. Finally, patients were scanned by multiple learners, but each set of LUS images was treated as independent in our analysis, thereby ignoring potential within-subject correlation for B-line counts. Image acquisition technique (probe location on chest wall, angle of insonation, timing during the respiratory cycle) has a substantial impact on image quality and thus B-line counts. Given the varied experience level of each learner and the fact that they scanned independently, we felt that, overall, between-learner variability in image acquisition technique would minimize the impact of the within-subject B-line correlation.

## Conclusion

After a limited structured training learners with no to little prior experience performing LUS were able to obtain high-quality images. However, B-line quantification of these images using a deep learning AI algorithm found only moderate-to-fair correlation when compared to semi-quantitative analysis by an expert. This data shows promise, but further development is needed before widespread clinical use.

## Data Availability

The datasets used and/or analyzed during the current study are available from the corresponding author on reasonable request.

## Abbreviations

LUS: Lung ultrasound
AHF: Acute heart failure
ED: Emergency departments
AI: Artificial intelligence
EM: Emergency medicine
NYHA: New York Heart Association
ICC: Intraclass correlation
CI: Confidence interval
